# Enhanced Hemocompatibility and Cytocompatibility of Stainless Steel

**DOI:** 10.1021/acsomega.4c01191

**Published:** 2024-04-16

**Authors:** Metka Benčina, Niharika Rawat, Domen Paul, Janez Kovač, Katja Lakota, Polona Žigon, Veronika Kralj-Iglič, Aleš Iglič, Ita Junkar

**Affiliations:** † Department of Surface Engineering, Joz̆ef Stefan Institute, Jamova 39, SI-1000 Ljubljana, Slovenia; ‡ Laboratory of Physics, Faculty of Electrical Engineering, University of Ljubljana, Tržaška 25, SI-1000 Ljubljana, Slovenia; § Department of Rheumatology, University Medical Centre Ljubljana, Vodnikova 62, SI-1000 Ljubljana, Slovenia; ∥ Laboratory of Clinical Biophysics, Faculty of Health Sciences, University of Ljubljana, Zdravstvena pot 5, SI-1000 Ljubljana, Slovenia; ⊥ Laboratory of Clinical Biophysics, Faculty of Medicine, University of Ljubljana, Vrazov trg 2, SI-1000 Ljubljana, Slovenia

## Abstract

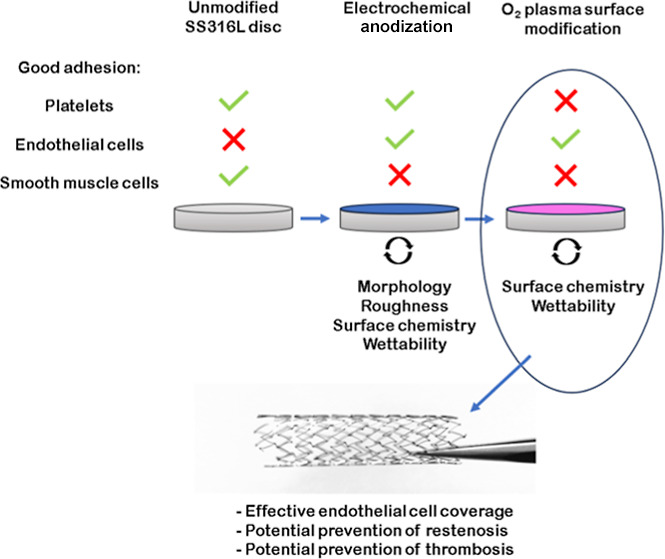

The present study introduces an advanced surface modification approach combining electrochemical anodization and non-thermal plasma treatment, tailored for biomedical applications on stainless steel grade 316L (SS316L) surfaces. Nanopores with various diameters (100–300 nm) were synthesized with electrochemical anodization, and samples were further modified with non-thermal oxygen plasma. The surface properties of SS316L surfaces were examined by scanning electron microscopy, atomic force microscopy, X-ray photoemission spectroscopy, and Water contact angle measurements. It has been shown that a combination of electrochemical anodization and plasma treatment significantly alters the surface properties of SS316L and affects its interactions with blood platelets and human coronary cells. Optimal performance is attained on the anodized specimen featuring pores within the 150–300 nm diameter range, subjected to subsequent oxygen plasma treatment; the absence of platelet adhesion was observed. At the same time, the sample demonstrated good endothelialization and a reduction in smooth muscle cell adhesion compared to the untreated SS316L and the sample with smaller pores (100–150 nm). This novel surface modification strategy has significant implications for improving biocompatibility and performance of SS316L in biomedical applications.

## Introduction

Vascular stents are mesh tubes used to treat narrowed, blocked, or weakened arteries. These medical devices are crucial in treating atherosclerosis, where arteries can narrow due to plaque buildup in an artery’s inner lining. After implantation, they act as scaffolding to prevent the artery from collapsing or becoming reoccluded, ensuring continuous blood flow to the heart muscle. This long-lasting support is essential for patients with significantly narrowed arteries, helping to alleviate symptoms such as chest pain and reduce the risk of heart attacks.

Stents are commonly constructed from biocompatible materials such as stainless steel (SS) 316L (SS316L), nickel–titanium (NiTi), or cobalt–chromium (CoCr) alloys since these materials exhibit high tensile strength and flexibility, characteristics that are essential for the durability and strength that a stent should provide over a long-time range. Despite intensive research, stents made of metal alloys still face several challenges. One significant concern is the risk of restenosis after stent implantation, which is called in-stent restenosis. This can occur due to neointimal hyperplasia, where the inner lining of the artery grows excessively around the stent.^1^ Another issue is stent-thrombosis, a rare but severe condition where a blood clot forms at the stent site,^2^ which could lead to a heart attack or stroke. Additionally, patients with metal stents typically require long-term antiplatelet therapy, which increases the risk of bleeding complications.

On the other hand, biodegradable stents, typically made from materials like polylactic acid, polyglycolic acid, or even magnesium alloys, are designed to dissolve within the body over time and are particularly valuable when only temporary vascular support is needed.^3^ Biodegradable stents reduce the risk of long-term complications, such as chronic inflammation or late thrombosis, and potentially eliminate the need for removal surgery. However, biodegradable stents, while avoiding long-term foreign body presence, face significant drawbacks compared to metallic stents. They are more likely to lead to early restenosis due to their temporary structural support as they degrade over time,^4^ contrasting with the more durable support offered by metallic variants. This reduced support can result in a quicker vessel patency loss. Additionally, the degradation process of biodegradable stents can sometimes lead to uneven support to the vessel wall, potentially resulting in complications like vessel recoil.^5^

SS grade 316L (SS316L) is a widely utilized material in the medical field due to its excellent structural properties, effectiveness in load bearing and fixation, chemical stability, and cost-effectiveness.^6−8^ It is used to manufacture medical devices, equipment, and surgical tools and remains among the most popular implant choices in various medical disciplines, including cardiovascular treatments, orthopedics, dentistry, and craniofacial surgery. In the case of vascular stents, SS316L is preferred for its corrosion resistance, strong mechanical strength, and good biocompatibility. SS includes elements like chromium, nickel, and molybdenum, which contribute to its resistance against the chloride-rich environments typical of human body fluids^9^ and, consequently, minimizes the risk of adverse reactions such as inflammation or allergic responses. However, despite its high biocompatibility, there is still a risk of ion-leaching, which can be a concern for patients with allergies.^10^

To enhance the biocompatibility of SS316L, various surface modification techniques are employed to optimize its performance for medical applications. Among these techniques, electrochemical anodization of SS has emerged as a promising approach to precisely tailoring the surface properties of SS, thereby significantly improving its corrosion resistance, antibacterial properties, and cytocompatibility.^11−14^ This process involves applying an electrical current to the SS, which is used as an anode in an electrolytic solution, forming an oxide layer on the surface, mostly with a nanoporous morphology with a tunable pore size. Benčina et al.^15^ review synthesizing procedures for preparing nanoporous SS materials mainly based on anodic oxidation. The resulting oxide layer exhibits improved biocompatibility, making it highly attractive for biomedical applications, including implants and medical devices, since the nanoscale topography created can significantly influence cellular interactions with the SS surface. For instance, Ni et al.^12^ prepared 316L SS samples with adjustable nanometer pit sizes (0, 25, 50, and 60 nm) through an anodization process in an ethylene glycol electrolyte solution containing 5 vol % perchloric acid. The anodized surfaces exhibited improved wettability, enhanced protein adsorption, and significantly facilitated the initial attachment and growth of human dermal fibroblasts for up to 3 days in culture. Hsu et al.^16^ showed that anodized 316L SS exhibits improved cell adhesion and coverage for osteoblast-like cells (MG-63), indicating its potential for promoting bone formation. Recently, Erdogan and Ercan^11^ showed that anodization of 316L SS surfaces resulted in the formation of nanodimples with controlled sizes, leading to increased surface area and altered surface chemistry with chromium oxide- and hydroxide-rich layers; these nanodimpled surfaces demonstrated enhanced osteoblast viability and function, along with reduced *Staphylococcus aureus* and *Pseudomonas aeruginosa* biofilm formation. Furthermore, Jang et al.^14^ prepared nanotextured SS 316L, developed through electrochemical etching, which demonstrated effective inhibition of bacterial adhesion (*Escherichia coli* and *S. aureus*), providing cytocompatibility, nontoxicity to mammalian cells, and improved corrosion resistance. In another study, Cherian et al.^17^ modified the surface of SS stents with nanoscale titania topography, reducing in-stent restenosis in rabbit iliac arteries for 8 weeks, highlighting the potential of this cost-effective modification for coronary applications without additional agents or polymers. These results prove that surface modification techniques can significantly improve SS bioperformance.

Despite its use in vascular stent production, there needs to be more comprehensive research regarding SS’s interaction with blood platelets, human coronary artery endothelial cells (HCAEC), and human coronary artery smooth muscle cells (HCASMC). We hypothesize that the modification of SS316L surfaces through an electrochemical anodization process, resulting in nanoporous structures, followed by oxygen plasma treatment, can significantly enhance the biocompatibility of SS316L. This enhancement is anticipated to foster favorable interactions with blood platelets and vascular cells. The surfaces were modified using an electrochemical anodization process, resulting in the creation of various nanoporous surfaces. Following the anodization process, the samples were subjected to oxygen plasma treatment, a low-pressure plasma technique well-recognized for its remarkable ability to modify the surface properties of materials. The primary aim of this study was to harness the potential of this combined approach to enhance the biocompatibility of SS316L, which is a critical aspect of its application in various biomedical devices and implants. Our investigations have unveiled the promising capabilities of this surface modification strategy. The treated SS316L surfaces exhibited improved biocompatibility, which is crucial for reducing the risk of adverse reactions and ensuring the longevity of biomedical implants. This surface treatment also improved the surface wettability, which facilitates the interactions between the material and biological fluids, thereby promoting optimal performance in physiological environments. Overall, the combination of electrochemical anodization and oxygen plasma treatment shows great promise in advancing the field of biomedical materials and implant technology.

## Experimental Section

### Materials and Methods

Ethylene glycol (Carlo Erba, for analysis), perchloric acid (Honeywell, ACS reagent 70%), acetone, and absolute ethanol. A 316-grade stainless steel (SS316L) rod (4 mm thick, High Performance Metals, Austria) was cut into specimens of discs with a diameter of 15 mm and a thickness of 5 mm by using a water jet cutter. One side of the disc surfaces underwent a sequential polishing process, starting from 400-grit and advancing to 3000-grit emery paper, ultimately achieving a mirror-like finish using diamond paste. Subsequently, the discs were subjected to ultrasonic cleaning in absolute ethanol to remove any residual particles or contaminants.

### Electrochemical Anodization

Electrochemical anodization was performed by a Voltcraft VSP 2653 in a Teflon vessel, as a cathode Pt foil (0.1 mm thick, Premion, 99.99%, metals basis) was used. SS316L discs were used as an anode. The distance between the electrodes was 1 cm. Before anodization, specimens were cleaned by sonicating with ethanol (70%) and distilled water for 10 min each to remove surface impurities and subsequently dried in air. 5% perchloric acid was prepared in ethylene glycol and used as an electrolyte. Two distinct voltages were employed: 40 and 60 V. Meanwhile, the synthesis duration was maintained consistently at 10 min for all experiments. After the synthesis, the samples were thoroughly rinsed and ultrasonicated in absolute ethanol for 10 min. Samples were kept in absolute ethanol for 2 h, dried under the stream of N_2_ and stored inside plastic containers tightly sealed with parafilm. The following samples were tested in further experiments: untreated SS316L (SS), plasma-treated SS316L (SS + P), SS316L anodized at 40 V (SS40), SS316L anodized at 60 V (SS60), and plasma-treated anodized samples (SS40 + P and SS60 + P).

### Plasma Treatment of SS316L Discs

The treatment of SS316L discs was performed in the in-house designed plasma reactor, as reported in ref (18) Briefly, the system was evacuated with a two-stage oil rotary pump (Edwards E2M80) with a nominal pumping speed of 80 m^3^/h. The discharge chamber was a Pyrex discharge tube with a length of 0.8 m and an inner diameter of 0.036 m. At the center of the glass tube, a coil of six turns was mounted and coupled with a radiofrequency (RF) generator (Advanced Energy CESAR 1310) via the matching network (Dressler VM 1500 W-ICP). Gaseous inductively coupled plasma was created with a RF generator operating at a fundamental frequency of 13.56 MHz and a maximum output power of 1 kW. Commercially available oxygen with ≥99.9% purity was leaked into the discharge chamber with mass flow controllers (Aera FC 7700 Advanced Energy). The pressure was measured with an absolute vacuum gauge (722A MKS Baratron). Each sample was mounted in the reaction chamber in the middle of the excitation coil. After the discharge chamber was evacuated to a base pressure below 1 Pa, 5 sccm of oxygen was continuously leaked into the reaction chamber. The pressure was 75 Pa. The samples were treated for 60 s. After the plasma treatment, the samples were stored inside a tightly closed container sealed with parafilm.

### Characterization

#### Scanning Electron Microscope Analysis

The morphology of the surfaces and interactions with platelets were analyzed with a scanning electron microscope (SEM) (JSM 7600F-JEOL) equipped with a field emission gun with an acceleration voltage of 15 kV.

#### Water contact angle Measurements

The wettability measurements of SS316L were performed with the Drop Shape Analyzer DSA-100 (Krüss GmbH, Hannover, Germany) by a sessile drop method to measure a static contact angle. The contact angle on the surface was analyzed within 30 min after the electrochemical anodization/plasma treatment, adding a 2.5 μL drop of deionized water on 8 different surface areas. Three measurements were performed for each sample, and the average value was calculated. The relative humidity was around 45%, and the operating temperature was 21 °C, which did not vary significantly during continuous measurements.

#### Atomic Force Microscopy Analysis

Topographic features of SS316L samples were examined by atomic force microscopy (AFM) (Solver PRO, NT MDT) in tapping mode in the air. Samples were scanned with the standard Si cantilever with a force constant of 22 N/m and at a resonance frequency of 325 kHz (the tip radius was 10 nm, and the tip length was 95 μm), and the scan rate was set to 1.3 Hz. Surface roughness was assessed through the roughness average (Ra), calculated from 20 × 20 μm^2^ images taken from 5 different measurements.

#### X-ray Photoelectron Spectroscopy

The X-ray photoelectron spectroscopy (XPS) analyses were carried out on a PHI-TFA XPS spectrometer produced by Physical Electronics Inc. Samples were put on the sample holder and introduced into the ultrahigh vacuum spectrometer. The analyzed area was 0.4 mm in diameter, and the explored depth was about 3–5 nm. Sample surfaces were excited by X-ray radiation from a monochromatic Al source at a photon energy of 1486.6 eV. XPS depth profiles were performed to obtain the in-depth concentration of elements. Ar ion sputtering with an ion energy of 1 keV was applied. The sputtering rate was approximately 2 nm/min.

#### Incubation of the Samples with Whole Blood

The procedure for assessing platelet adhesion and activation on the samples was conducted as follows: before whole blood incubation, samples were cleaned with ethanol, dried, and incubated with whole blood taken by vein puncture from a healthy human donor. The blood was drawn into 9 mL tubes coated with trisodium citrate anticoagulant. Afterward, the fresh blood (250 μL) was incubated with samples in 24-well plates for 45 min at room temperature. After incubation, 250 μL of phosphate-buffered saline (PBS) was added to the whole blood. The blood with PBS was removed, and the SS surface was rinsed 5 times with 250 μL of PBS to remove weakly adherent platelets. Adherent cells were subsequently fixed with 250 μL of a 0.5% GA (glutaraldehyde) solution for 15 min at room temperature. Afterward, the surfaces were rinsed with PBS and then dehydrated using a graded ethanol series (50, 70, 80, 90, 100, and again 100 vol % ethanol) for 5 min, and in the last stage (100 vol % ethanol) for 15 min. Then, the samples were placed in a critical point dryer, where the solvent was exchanged with liquid carbon dioxide. By increasing the temperature in the drier, the liquid carbon dioxide passes the crucial point at which the density of the liquid equals the density of the vapor phase. This drying process preserves the natural structure of the sample and avoids surface tension, which could be caused by regular drying. The dried samples were subsequently coated with gold/palladium and examined using SEM. The test was performed in triplicate; only representative images are shown in this paper. Tests were performed by following the Declaration of Helsinki and approved by Slovenia’s Ethics Committee (number of the approval 56/03/10).

#### Interactions with Endothelial and Smooth Muscle Cells

HCAEC were purchased from Lifeline Cell Technology (Frederick, MD, USA), and HCASMC were purchased from ProVitro AG (Berlin, Germany). HCAEC and HCASMC were plated into 75 cm^2^ flasks (TPP, Trasadingen, Switzerland) at 37 °C in a humidified atmosphere at 5% CO_2_ and grown in VascuLife EnGS endothelial medium complete kit (Frederick, MD, USA) and Smooth muscle cell growth medium FCS-kit (ProVitro AG, Berlin, Germany), respectively, as advised by the manufacturer’s instructions. For experiments, subconfluent cell cultures were used between passages 4 and 6. HCAEC and HCASMC were seeded onto untreated and modified SS316L discs. The cells on sample materials (15 mm^2^) were placed into 12-well plates at a density of −20 × 10^3^ cells per cm^2^ and grown for 2 days; the test was conducted in triplicates. Staining with fluorescein phalloidin (Molecular Probes, Thermo Fisher Scientific) was performed following the manufacturer’s instructions. Briefly, cells were washed 2 times for 3 min with PBS, pH 7.4, fixed in a 3.7% formaldehyde solution for 10 min, and washed 3 times for 3 min with PBS at room temperature. Cells were incubated in 0.1% Triton X-100 for 4 min and then washed with PBS 3 times for 3 min. Dye stock was diluted 1:40 in PBS with 1% BSA and applied to HCAEC and HCASMC for 30 min. The final washing steps were performed 3 times for 3 min with PBS. DAPI (4′,6 diamidino 2 phenylindole) staining (Molecular Probes, Thermo Fisher Scientific) followed the manufacturer’s instructions. Briefly, samples were incubated with 300 nM DAPI in PBS for 5 min and washed with PBS for 3 min at room temperature. SlowFade reagent (Thermo Fisher Scientific, USA) was applied to HCAEC and HCASMC (1 drop), and a coverslip was fixed on top with clear nail polish. Slides were examined and stored in the dark at 4 °C. Images were generated using the Nikon Eclipse E400 fluorescent microscope and a digital camera (Nikon Instruments, Dusseldorf, Germany). Analysis was performed with a Nikon ACT-1 imaging software, and representative images were presented.

## Results and Discussion

### Surface Morphology

**Figure 1:**
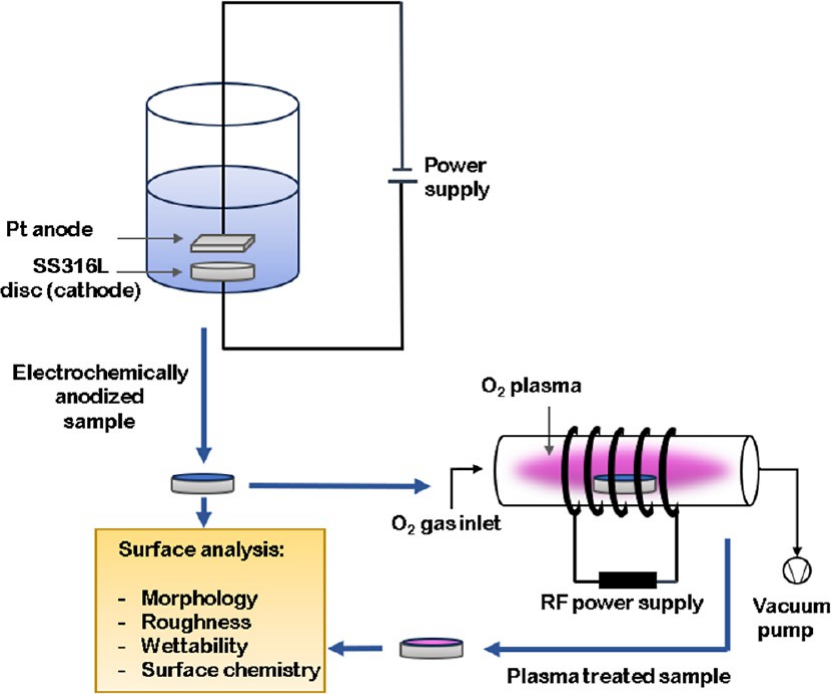
Schematic representation of electrochemical anodization and oxygen plasma treatment of SS316L discs.

The samples underwent preparation through electrochemical anodization using an electrolyte containing perchloric acid, in which SS discs were used as the anode and platinum (Pt) as the cathode. Following anodization, selected samples were additionally treated with non-thermal plasma. These methods are depicted schematically in Figure 1.

**Figure 2:**
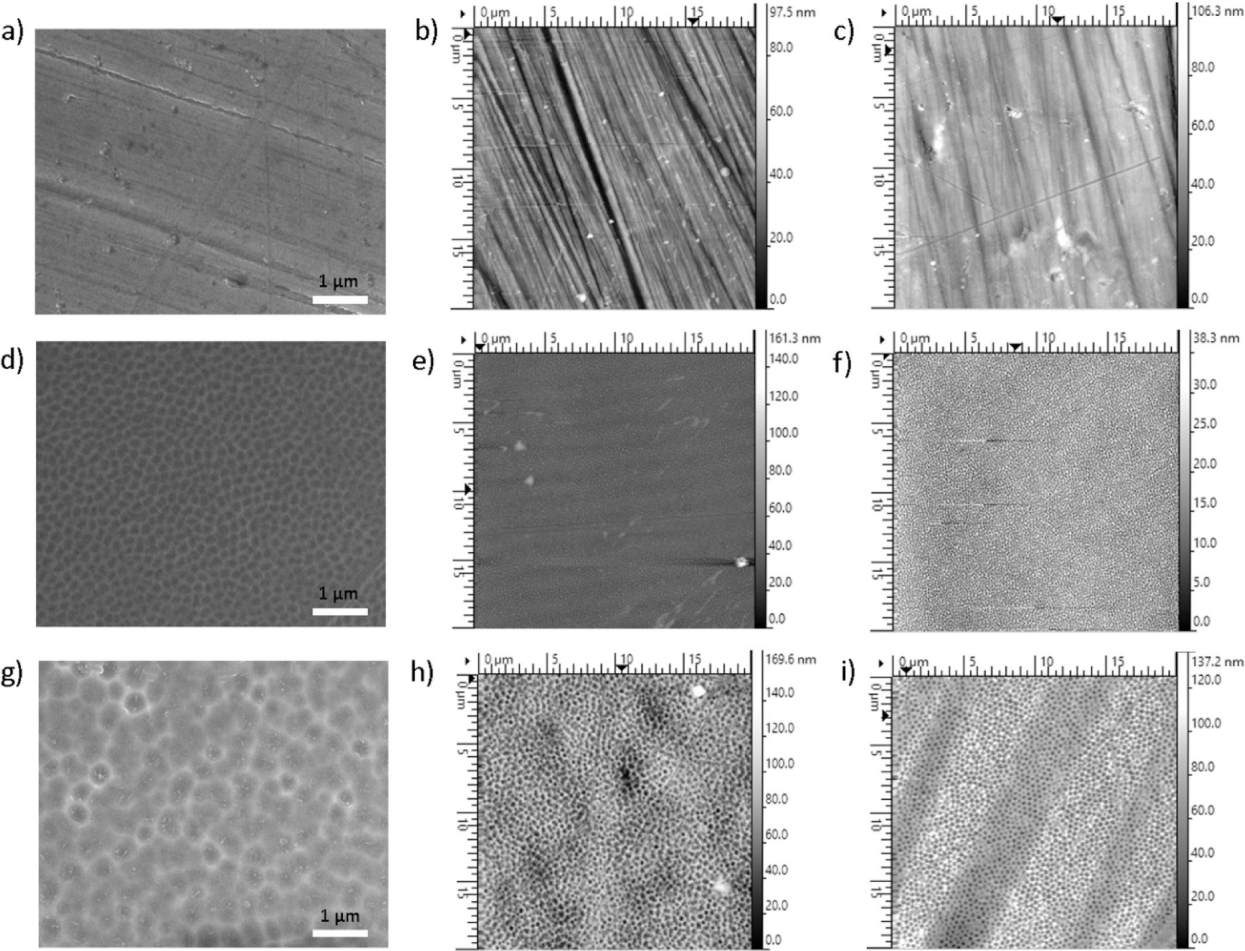
SEM and AFM images of untreated and modified SS316L; (a) SEM image of SS, AFM image of (b) SS, and (c) plasma-treated SS; (d) SEM image of SS40, AFM image of (e) SS40, and (f) plasma-treated SS40 + P; and (g) SEM image of SS60, AFM image of (h) SS60, and (i) plasma treated SS60 + P.

The results of this study reveal a significant correlation between the anodization voltage and the formation of nanopores on the surface of SS316L (Table 1). On the surface of the SS316L sample (SS), which did not undergo electrochemical anodization and plasma treatment, the morphology characteristics of the polishing process can be observed (Figure 2). By systematically varying the anodization voltage, distinct nanopore diameters were achieved, underscoring the tunability of the anodization process for tailoring surface characteristics. Samples anodized at 40 V (SS40) exhibited a nanoporous morphology, with nanopore diameters varying from 100 to 150 nm, while those anodized at 60 V (SS60) resulted in even larger nanopores, with diameters spanning roughly 150–300 nm. SEM analysis indicated no morphological alterations following plasma treatment; consequently, the results of plasma-treated samples are not included in the presented data.

**Table 1:** Elemental Surface Composition in at % Determined by XPS Analysis, Pore Diameter, Average Surface Roughness, and Wettability of Untreated and Modified SS316L

sample	C 1s	O 1s	Cr 2p_3/2_	Mn 2p_3/2_	Fe 2p_3/2_	Ni 2p_3/2_	Mo 3d	pore diameter (nm)	Ra (nm)
SS	63.0	28.2	3.2	1.2	3.5	0.6	0.2		6.3 ± 0.3
SS + P	24.1	55.2	5.1	1.1	12.4	2.0	0.1		5.0 ± 0.5
SS40	58.2	26.4	5.0	0.9	8.1	0.8	0.6	100–150	4.3 ± 0.4
SS40 + P	16.3	55.0	6.7	0.4	18.1	3.3	0.2	100–150	4.3 ± 0.7
SS60	53.0	32.1	7.8	1.1	5.6	0.3	0.1	150–300	15.0 ± 0.6
SS60 + P	19.4	56.9	6.3	1.5	15.4	0.3	0.2	150–300	14.5 ± 0.6

**Figure 3:**
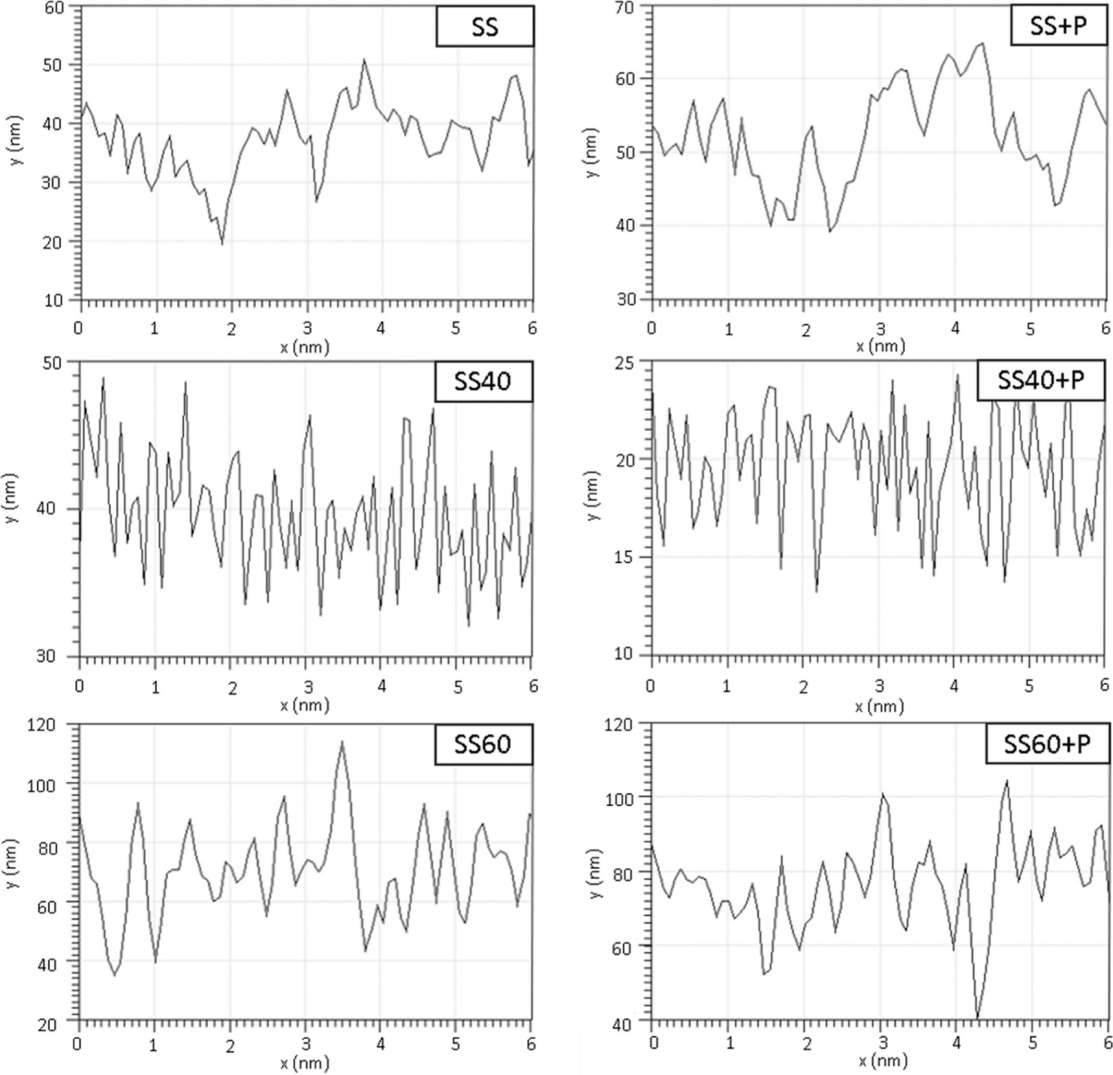
Height profiles of untreated (SS) and modified SS316L.

Topography images of samples obtained by AFM analysis (Figure 2) are in agreement with the SEM results; it can also be observed that plasma treatment does not alter the surface morphology of SS, SS40, and SS60. The surface roughness (height profiles presented in Figure 3) can further confirm this; in the case of SS and SS + P, about 6 and 5 nm Ra were measured, respectively (Table 1). A slight reduction of surface roughness was further observed for the SS40, Ra for SS, and SS40 + P, which was about 4 nm. However, a contrary trend was observed for SS60, where the Ra concentration increased to about 15 nm (Table 1). More pronounced and bigger nanopores on SS60 compared to SS40 can be seen from AFM analysis. The increase in size of the nanopores on SS60 compared to SS40 is attributed to the modification of a key synthesis parameter, precisely the voltage. Increasing the voltage during the electrochemical anodization process of SS leads to an increase in the diameter of the nanopores formed on the surface, as shown elsewhere.^11,15,19,20^ The correlations between the anodization parameters and the surface morphology of SS have been described by Benčina et al.^15^ The possible explanation for the larger pore diameter with increasing anodization voltage could lie in increased electric field strength across the anodic oxide layer, which accelerates the ion migration and dissolution processes at the electrolyte/oxide interface.

### Surface Chemistry and Depth Profile Analysis

**Figure 4:**
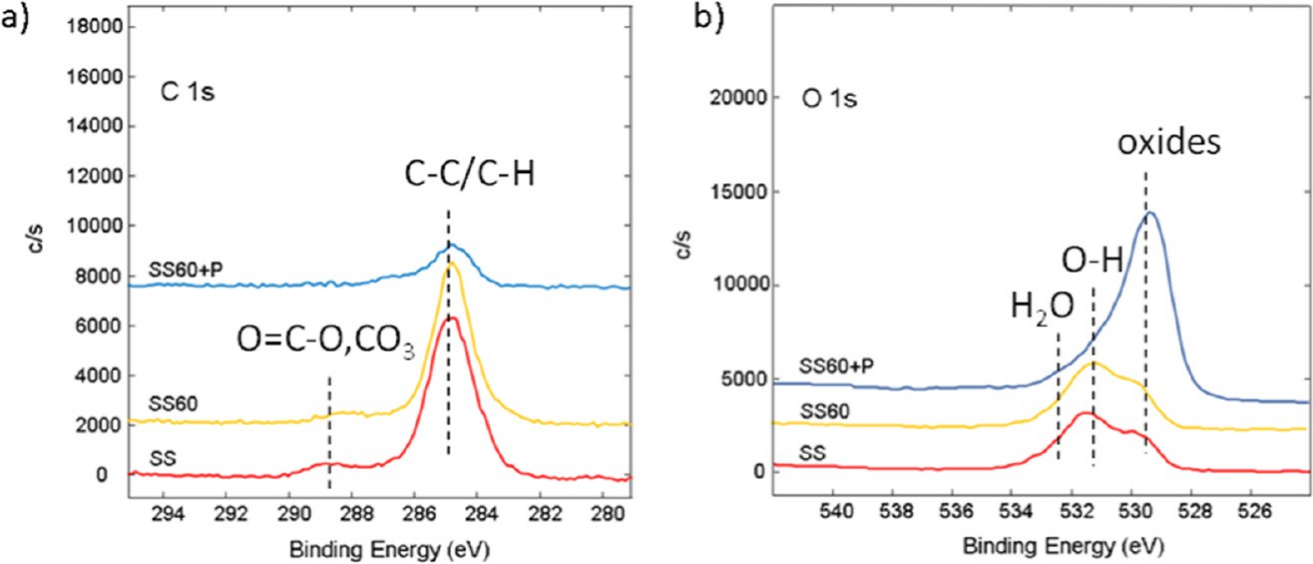
High-resolution XPS spectra of (a) C 1s and (b) O 1s for untreated (SS) and modified SS316L.

The surface composition of the untreated and modified SS316L samples was determined by XPS, as shown in Figure 4 and Table 1. The elements on the surface of SS samples are carbon, oxygen, iron, chromium, and nickel, with trace amounts of molybdenum. The amount of oxygen on the surface of untreated and anodized SS316L samples is similar; however, the increased oxygen concentration is observed on the surface of plasma-treated samples (SS + P, SS40 + P, and SS60 + P). In addition, decreased carbon concentration on plasma-treated surfaces is observed, which can be ascribed to the efficacious cleaning effects of the plasma treatment, wherein the removal of contaminants from the surface is well-documented.^21,22^ Anodized samples also exhibit higher concentrations of chromium, iron, and nickel on the surface than untreated SS. The concentration of chromium and iron is even higher for samples additionally treated by plasma (SS + P, SS40 + P, and SS60 + P). Also, the amount of nickel increases with anodization and/or plasma treatment. Removing carbon from the top surface can enhance surface energy^23^ and promote better wetting characteristics. This is crucial in applications where surface wetting is essential, such as biomedical devices, where improved wetting can lead to better biocompatibility and a reduced risk of biofilm formation.^24^ This makes plasma treatment a valuable technique for enhancing the performance and functionality of materials in diverse industrial and research applications.

High-resolution XPS was applied to evaluate chemical bonds. The high-resolution C 1s and O 1s spectra of SS untreated and modified (SS60 and SS60 + P) samples are presented in Figure 4, as similar spectra were observed for all–untreated, anodized, and anodized/plasma-treated samples. The C 1s spectrum mainly consists of the peak at around 285.0 eV, which corresponds to carbon–carbon (C–C) and carbon–hydrogen (C–H) bonds that could be found on SS due to contamination (Figure 4a). There is also a small peak at 288.9 eV, probably related to the presence of the O=C–O and/or CO_3_ bonds from surface contamination. After plasma treatment (SS60 + P), the C 1s peak intensity is reduced, suggesting the removal of organic residues on the surface due to plasma etching.

**Figure 5:**
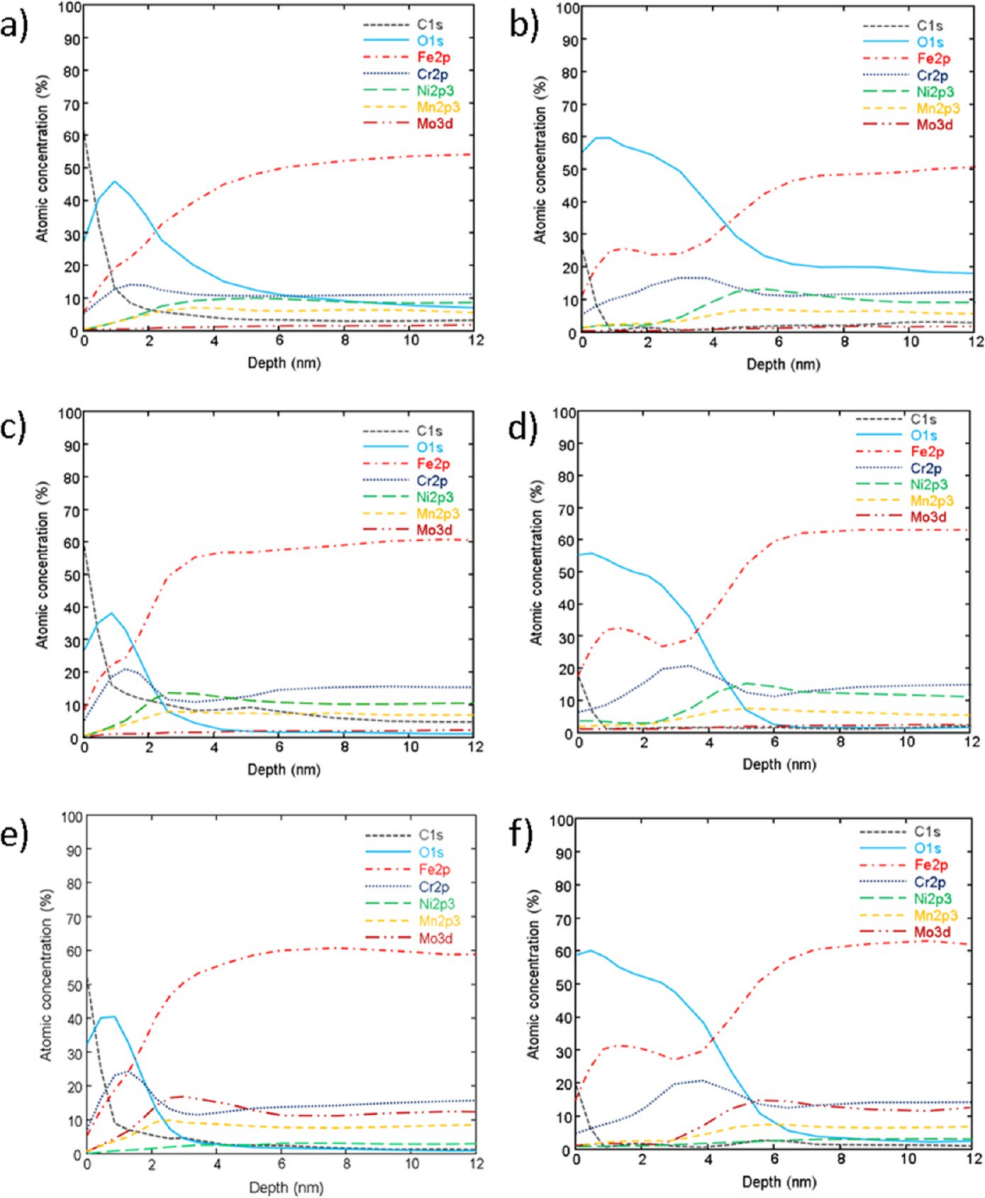
Depth profile analysis of untreated and modified SS316L; (a) SS, (b) SS + P, (c) SS40, (d) SS40 + P, (e) SS60, and (f) SS60 + P.

The O 1s spectra for SS and SS + P revealed distinct peaks indicative for varied oxygen-containing functional groups (Figure 4b), like a peak at 529.5 eV related with oxygen atoms in the oxide structure and peaks at 531.7 and 532.5 eV. After plasma treatment, the O 1s peak at 529.5 eV increased significantly, suggesting the prevalence of metal oxides, such as Cr_2_O_3_, Fe_2_O_3_, MnO, MoO_3_, and NiO. This is observed for all plasma-treated surfaces, and it is well correlated with the higher content of Cr, Fe, and Ni on the surface (Table 1). These findings could also be correlated with thicker metal oxide layer, as corroborated by the prevailing scientific literature^25−27^ as well as our results from depth profile analysis (Figure 5). Peaks at higher binding energies, notably around 531.2 and 532.5 eV, are discerned and speculated to correspond to hydroxyl groups (OH−) and adsorbed water molecules, respectively.^28,29^

The depth profile analysis from the provided data indicates notable differences between untreated, anodized, and plasma-treated SS316L samples (Figure 5). Initially, carbon is present on the surface layers of both untreated and anodized SS316L samples, indicative of organic contamination or native oxide layers typically found on SS surfaces. Conversely, plasma-treated surfaces show a pronounced increase in the oxygen layer thickness, suggesting the formation of a more substantial oxide layer, primarily composed of iron and chromium oxides, indicative of enhanced surface oxidation due to plasma treatment.

For the untreated (SS) and anodized SS316L samples (SS40 and SS60), the oxide layer thickness is approximately 2 nm, suggesting a relatively thin native oxide film. This thickness is consistent with the natural passive film typically found on SS surfaces, which forms spontaneously upon exposure to air. In contrast, all plasma-treated samples, specifically SS + P, SS40 + P, and SS60 + P, exhibit a notable alteration in surface characteristics, with the oxide layer thickness approximately doubling to about 4 nm. This increase indicates enhanced oxide growth, which is a direct consequence of the plasma treatment. This process involves the material surface being bombarded with high-energy ions, electrons, and neutral species generated from gaseous plasma, fundamentally altering the surface chemistry and topography of the SS surfaces. When these surfaces are exposed to an oxygen-rich plasma, the resultant environment is highly reactive, characterized by an abundance of oxygen ions, radicals, and atoms. This increased interaction between the oxygen plasma and SS leads to a more accelerated and aggressive oxidation process compared to conventional atmospheric exposure. The generation of reactive oxygen species in the plasma facilitates deeper penetration and higher reactivity than under ambient conditions, thereby significantly enhancing the growth of the oxide layer. This phenomenon is primarily attributed to the plasma’s ability to supply a consistent and elevated flux of reactive species, which interact with the surface to produce a denser, more comprehensive oxide layer, thereby improving the surface’s protective qualities and potentially its corrosion resistance properties.

The depth profiles for iron and chromium oxides show significant variations between the samples. The untreated SS and SS40 samples exhibit an increase in iron oxide at the surface, suggesting that the anodization process at lower voltages may preferentially oxidize iron components in the alloy. In contrast, the SS60 sample shows a higher surface concentration of chromium relative to iron oxide, which is advantageous for corrosion resistance as chromium oxides form more stable and protective layers compared to iron oxides.

After plasma treatment, there is a noticeable shift with iron oxides becoming more dominant on the surface, particularly for SS40 + P and SS60 + P samples. This suggests that the plasma process may alter the surface chemistry, preferentially removing or modifying the chromium oxide layer and enhancing the presence of iron oxide on the surface. The depth profiles also indicate a reduction of other oxides such as Cr, Ni, Mo, and Mn from the top surface into deeper regions of the sample, suggesting a redistribution of these elements within the oxide layer due to plasma treatment. This redistribution could potentially affect the surface layer’s long-term corrosion resistance and mechanical properties.

### Surface Wettability

**Figure 6:**
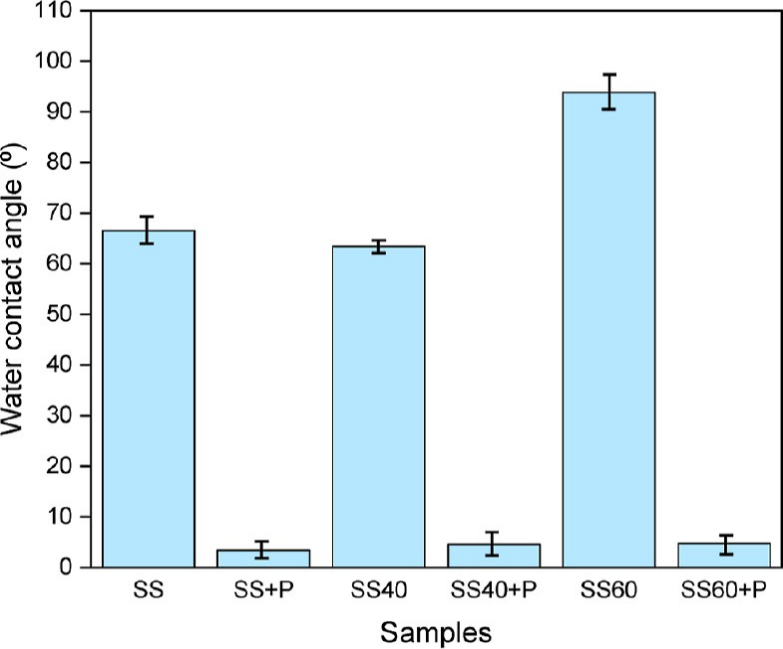
WCA analysis of untreated and modified SS316L.

In the current investigation, untreated SS demonstrates a hydrophilic nature with a water contact angle (WCA) of about 66° (Figure 6). Notably, an increase in nanopore diameter corresponds to an increased WCA, reaching approximately 94° for SS60, indicative of hydrophobic behavior. This observed trend aligns consistently with previous studies.^30^ Also, Chen et al.^31^ developed superhydrophobic stainless surfaces with hierarchical micro/nanostructures using a nanosecond laser process and by so significantly enhanced water repellency due to the air layer formed between water and surface. In the present study, a similar mechanism may apply where the enhanced hydrophobicity is attributed to the formation of larger nanopores. However, when plasma treatment is used, there is a significant change in the wettability of all SS316L samples; the WCA drops to less than 5°. Thus, superhydrophilic behavior is observed for SS + P, SS40 + P, and SS60 + P. This highlights how effective plasma treatment alters the wetting properties of SS316L surfaces. The reason for this could be that oxygen plasma treatment enhances surface wettability by altering the surface chemistry, primarily through introducing and activating hydrophilic functional groups, such as hydroxyls, or by removing surface contaminants. When SS undergoes oxygen plasma treatment, its surface is enriched with polar functional groups such as hydroxyl.^32^ These groups are known to significantly increase the surface’s hydrophilicity,^33^ enhancing its ability to interact and bond with water molecules, thereby improving its wettability. On the other hand, plasma treatments involve bombarding the surface with high-energy ions, which can remove contaminants and organic compounds, including carbon-based residues.^34−36^ By reducing the carbon concentration on the surface of SS316L, which has also been confirmed by XPS analysis, the plasma treatment decreases the surface’s hydrophobicity.

**Figure 7:**
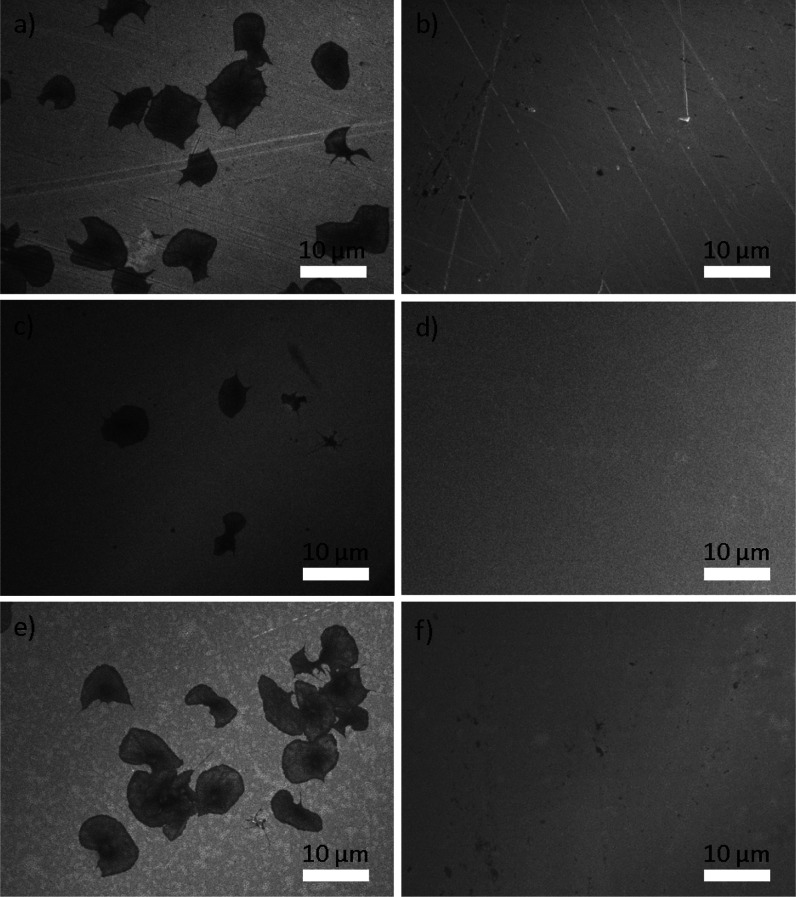
SEM images of platelets on the surface of untreated and modified SS316L; (a) SS, (b) SS + P, (c) SS40, (d) SS40 + P, (e) SS60, and (f) SS60 + P.

### Interactions with Platelets, HCAEC, and HCASMC

Understanding how platelets interact with various surfaces is essential in crafting biocompatible coatings to minimize the occurrence of thrombosis.^37^ Stents may induce different responses from blood platelets, including adhesion, activation, and aggregation.^38−40^ Adhesion involves platelets adhering to the surface, activation refers to changes in platelet shape and function, and aggregation involves the clumping together of platelets.^41−43^ These interactions can trigger complex biological responses, including blood clots or thrombus formation. Researchers are exploring numerous approaches for stent surface modifications to finely tune platelet interactions, optimize biocompatibility, and ultimately enhance the overall efficacy and safety of these medical devices in cardiovascular applications. For instance, to inhibit platelet adhesion and aggregation on SS316L SS, Yang et al.^44^ developed an antithrombogenic coating based on monoethylene glycol silane, which demonstrated a significant (>90%) reduction in platelet adhesion and aggregation during exposure to whole human blood. Similarly, Carmagnola et al.^45^ modified the surface of SS316L stents with a layer-by-layer coating, employing poly(styrenesulfonate)/poly(diallyldimethylammonium chloride) and heparin, and inhibited platelet adhesion and activation.

Interaction with whole blood was also studied to evaluate platelet adhesion and activation, which are highly connected with surface properties. In our study, the surfaces of SS316L samples were subjected to modification rather than coating, which reduces the risk of delamination and poor coating stability. The adhesion of platelets was mostly pronounced on SS and SS60, as presented in (Figure 7). On these surfaces, platelets displayed robust adhesion, appearing abundant and widely spread, while the presence of filopodia was limited. This suggests a heightened activation level among the platelets. Only a few platelets were observed on the surface of SS40, with these platelets appearing spread out and dendritic in morphology. However, platelets were not observed on all plasma-treated surfaces (SS + P, SS40 + P, and SS60 + P). The adhesion and heightened activation of platelets observed on untreated SS and SS60 suggest a potential tendency for platelet-related issues such as thrombosis, which could be a concern in medical device applications. On the other hand, the limited adhesion and dendritic morphology of platelets on SS40 imply a lower risk of thrombotic events. This surface treatment might benefit medical devices where minimizing platelet activation and adhesion is crucial, such as in the design of blood-contacting implants. Notably, the absence of platelets on plasma-treated samples (SS + P, SS40 + P, and SS60 + P) is particularly promising. Plasma treatment has shown effectiveness in preventing platelet adhesion, and this could be advantageous in medical applications where maintaining a nonthrombogenic surface is essential, for instance, in vascular stents or other implantable medical devices. Therefore, it can be concluded that the morphology of SS316L surfaces does not influence the adhesion of platelets as much as the chemistry and/or wettability. Plasma-treated surfaces (SS + P, SS40 + P, and SS60 + P) are super hydrophilic and have lower concentrations of carbon and higher concentrations of oxygen on the top surface compared to SS, SS40, and SS60 samples. Surface chemistry is indeed a crucial factor in influencing platelet behavior. Similarly, Phan et al.^46^ suggest that coating of SS316L with trimethylsilane followed by NH_3_/O_2_ plasma treatment inhibits platelet activation, among others, through the generation of NO and other N- and O-containing chemical groups on the SS316L surface, which mimic the inhibitory effects of NO on platelet adhesion and activation. However, surface morphology should not be neglected, and synergistic effects should probably be considered.

Interactions between SS and HCAEC and HCASMC can play a crucial role in the biocompatibility and performance of medical devices or implants. The behavior of these cells on SS surfaces is significant for applications such as vascular stents or other cardiovascular implants. The response of HCAEC is vital, as endothelialization of implant surfaces is desirable to promote blood compatibility and prevent thrombosis. Favorable interactions would involve cell adhesion, spreading, and proliferation, contributing to the formation of a functional endothelial layer. On the other hand, the behavior of HCASMC is also critical; while controlled adhesion and proliferation of smooth muscle cells are desirable for tissue healing and integration, excessive growth can contribute to restenosis, a complication in vascular interventions.

**Figure 8:**
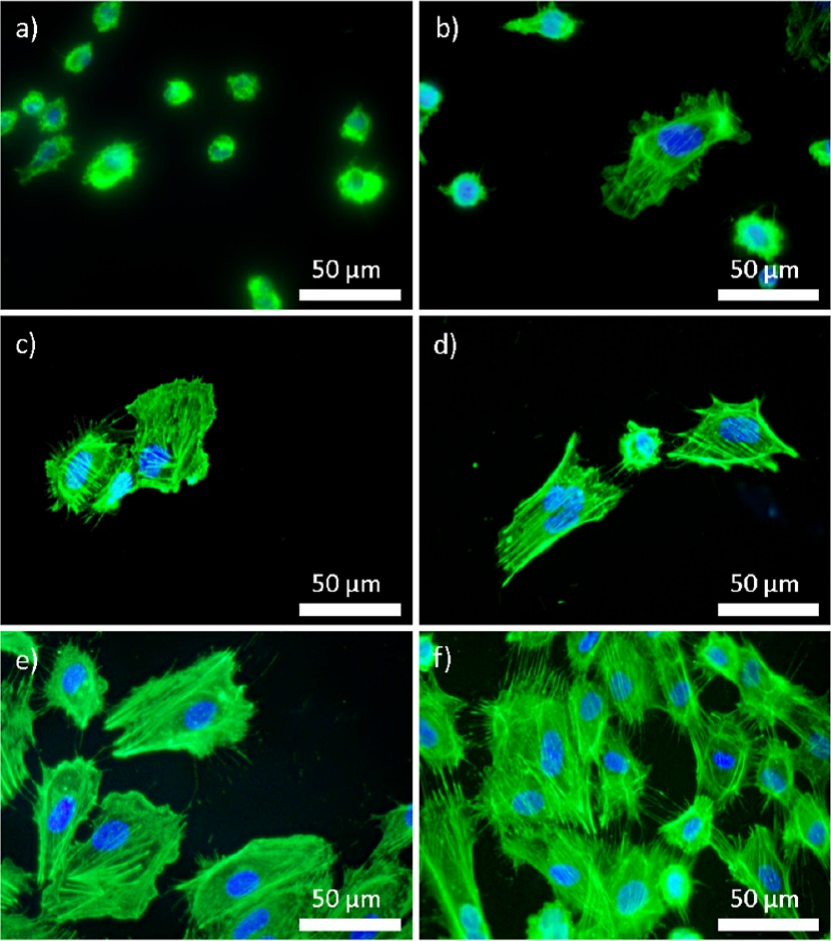
HCAEC on the surface of untreated and modified SS316L; (a) SS, (b) SS + P, (c) SS40, (d) SS40 + P, (e) SS60, and (f) SS60 + P.

**Figure 9:**
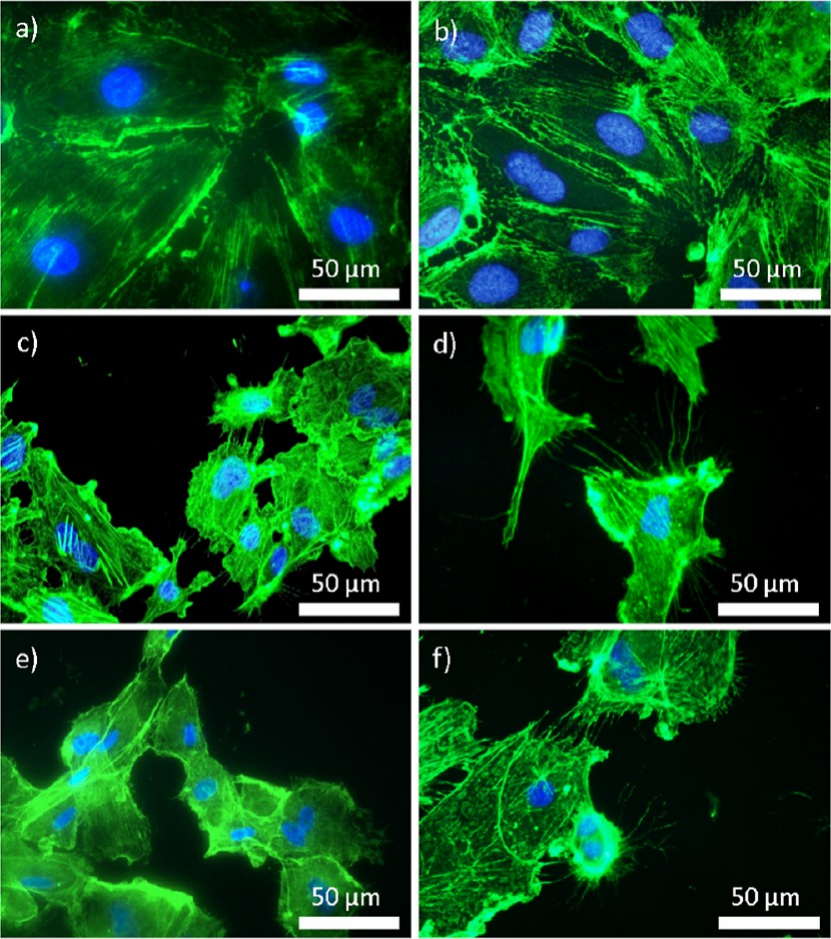
HCASMC on the surface of untreated and modified SS316L; (a) SS, (b) SS + P, (c) SS40, (d) SS40 + P, (e) SS60, and (f) SS60 + P.

The present study showed that HCAEC exhibits poor adhesion to surfaces such as SS and SS + P, as evidenced by the cell’s rounded shape and membrane blebbing, indicators of stress or suboptimal cell health (Figure 8). HCAEC attaches to the SS40 and SS40 + P; however, the surface of the samples is not fully covered by the cells, indicating a distinct interaction with these particular substrates. The most favorable performance is observed on the SS60 and SS60 + P surfaces, where cells adhere better but adopt an elongated form, signifying healthy engagement and psychological adaptation to the substrate. Surface topography is thus an important characteristic that influences cell behavior. The observation that HCAEC show improved adhesion and spreading on SS60 and SS60 + P surfaces (nanopore diameter of 150–300 nm) suggests that these nanopore sizes are optimal for mimicking the native cell environment, thereby enhancing cell–material interactions. Based on surface roughness, the results also suggest a differential adhesion behavior of HCAEC and HCASMC (Figure 9). Specifically, HCAEC prefer surfaces with increased roughness, as observed in the SS60 and SS60 + P samples (Figure 9e,f). Conversely, HCASMC display a contrasting affinity, demonstrating a greater propensity for adhesion to smoother surfaces, such as SS and SS + P. Ideally, the proliferation of HCASMC should be minimized relative to HCAEC to prevent issues such as neointimal hyperplasia, which can lead to stent restenosis. In the observed study, HCASMC demonstrate limited adhesion to substrates such as SS40, SS40 + P, SS60, and SS60 + P, indicating a potential mismatch between these surfaces and the cells’ physiological preferences. Furthermore, the appearance of membrane blebs is noted, which is a sign of cellular distress or apoptosis.

The results indicate that the optimized morphology and the beneficial effects of plasma treatment (altered surface chemistry, wettability) offer a viable approach to selectively enhancing endothelial cell growth while inhibiting smooth muscle cell proliferation. Various factors, including cell size, cell membrane curvature and stress, biochemical interactions, cellular signaling, and extracellular matrix deposition, can influence the interaction between HCAEC/HCASMC and nanoporous surfaces. The results of the present study suggest that HCASMC may favor less textured environments for adhesion. A similar effect of nanostructured surfaces on cell interactions as in the present study has also been shown by the research of Ni et al.;^47^ hUVEC cells readily adhere to porous anodic alumina (PAA) with a pore size of 75 nm compared to PAA with smaller pore sizes (25 nm), indicating the enhanced biological activity of PAA with larger pore sizes in promoting cell adhesion. The improved adhesion of cells on surfaces with larger nanopores can be attributed to several key factors. First, larger pores can facilitate a more extensive protrusion of the cell membrane features into the nanopores, enhancing cell adhesion through increased mechanical engagement. Additionally, these larger nanopores offer topographical features that mimic the naturally occurring extracellular matrix, further aiding in extensive cell attachment and spreading. The surface of unmodified SS in this study may lack the biological mimicry necessary for optimal endothelial cell interaction and attachment. To sum up, it has been shown that plasma-treated and anodized samples (SS40 + P and SS60 + P) show improved cell responses over just plasma-treated samples (SS + P) due to the combined effects of anodization and plasma treatment (improved morphology and wettability). This synergy enhances surface properties, promoting endothelial cell adhesion while reducing the adhesion of smooth muscle cells and platelets.

## Conclusions

The present study focuses on the surface modifications of SS (SS316L) through electrochemical anodization and non-thermal plasma treatment to determine the effect of altered surface properties on interaction with blood platelets and human coronary stents. The electrochemical anodization resulted in the formation of nanostructured surfaces, while additional plasma treatment increased the amount of oxygen on the surfaces. Therefore, after plasma treatment (indicated by SS + P, SS40 + P, and SS60 + P), there was a significant increase in the thickness of the oxide layer and also an alteration of wettability; plasma-treated surfaces were superhydrophilic. Surface modifications, in turn, substantially influenced cell adhesion behavior. Notably, the heightened hydrophilicity observed in samples subjected to oxygen plasma treatment has the potential to inhibit platelet adhesion. Also, a rougher surface, exemplified by SS60 and SS60 + P, can facilitate superior spreading and adhesion of endothelial cells compared to their smoother counterparts (SS and SS + P). In contrast, a higher count of smooth muscle cells was observed on the comparatively smoother surfaces (SS and SS + P). These findings clearly demonstrate the significant influence of surface topography on the selective adhesion and activity of different cell types.
